# Does Perceived Competence Mediate between Ball Skills and Children’s Physical Activity and Enjoyment?

**DOI:** 10.3390/children8070575

**Published:** 2021-07-04

**Authors:** Tao Zhang, Joonyoung Lee, Lisa M. Barnett, Xiangli Gu

**Affiliations:** 1 Department of Kinesiology, Health Promotion and Recreation, University of North Texas, Denton, TX 76203, USA; Joonyounglee@my.unt.edu; 2 Institute for Physical Activity and Nutrition, School of Health & Social Development, Deakin University, Geelong, VIC 3125, Australia; Lisa.barnett@deakin.edu.au; 3 Department of Kinesiology, University of Texas at Arlington, Arlington, TX 76019, USA; Xiangli.Gu@uta.edu

**Keywords:** ball skills, perceived competence, physical activity, enjoyment, children, elementary school

## Abstract

The major purpose of this study was to examine the potential mediating role of perceived motor skill competence on relationships between actual ball skills and children’s physical activity (PA) and PA enjoyment. A total of 294 students (*M*_age_ = 10.96 ± 0.76; 51.7% boys) from three elementary schools completed validated questionnaires assessing their perceived competence, self-reported PA, and PA enjoyment. Students’ actual ball skills (i.e., basketball, overhand throwing, striking) were measured by PE Metrics^TM^. Correlation analyses showed positive relationships among the study variables (*rs* ranging from 0.12 to 0.56). The structural equation modeling (SEM) analyses demonstrated that the mediation model produces a goodness-of-fit to the data: *χ*^2^/df = 52.03/32; CFI = 0.96; NFI = 0.90; IFI = 0.96, RMSEA = 0.05, SRMR = 0.04. Path coefficients suggested that actual ball skill competence was strongly associated with perceived competence (*β* = 0.36, *p* < 0.01), which in turn significantly predicted PA (*β* = 0.29, *p* < 0.01) and PA enjoyment (*β* = 0.35, *p* < 0.01). The findings highlight that ball skills significantly impact students’ perceived competence, positively and indirectly affecting their PA and PA enjoyment. This study provides empirical evidence that recommends intervention strategies aimed at fostering elementary school students’ PA and PA enjoyment.

## 1. Introduction

Regular physical activity (PA) has positive effects on physical, cognitive, and psychosocial health across the lifespan [1,2]. Nevertheless, school-aged children are not engaging in the suggested 60 min daily of moderate-to-vigorous PA (MVPA; [3]). Stodden et al. [4] conceptualized a theoretical model which suggested that as young children engaged in PA they developed their motor competence (fundamental motor skills [FMS]; proficiency in performing running, kicking, and throwing) and then as their motor competence developed, their PA participation increased. As such, the model is cyclical and reciprocal, and the relationship between motor competence and PA was theorized to strengthen over developmental time. Empirical evidence since 2008 suggests that actual motor competence among school-aged children is positively linked to vigorous engagement in PA [5,6]. Additionally, longitudinal evidence has shown that being a skilled child is associated with being a more active adolescent [7,8]. Review evidence reports a positive relationship between different FMS domains (both locomotor and ball skills) and PA during childhood to adolescence [9,10]. Although some studies have reported locomotor skill competence to be a predictor of PA [11,12,13], longitudinal relationships with PA have been shown in other studies regarding ball skill competence (also called object control skill) and not locomotor skill competence [7,14,15]. Considering the developmental model of Stodden et al. [4], the age of children in the sample is likely to impact whether locomotor or ball skill competence is a predictor of PA. In children of middle childhood (around 8 to 10 years), object control skill may be a salient predictor of PA, as these skills are required in many games and sports for this age group. The long-term effect of ball skill competence in PA could be associated with the skills (e.g., dribbling, striking, throwing) required to engage in moderate/vigorous sports training and competition in adolescence [7]. This may indicate that developing actual ball skill competence should be considered essential as a target to promote PA in school-aged children [7,15].

Perceived competence has also been linked to increased self-worth and motivation to engage in PA [16,17]. Perceived competence is defined as an individual’s beliefs or predictions toward their capabilities and abilities, which is an essential determinant of achievement-related behaviors and actions [18]. Recently, perceived competence has been considered as a more crucial personal factor than actual FMS competence for increasing the motivation among school-aged children to engage in different types of PA [16,19]. Furthermore, the findings of longitudinal research has supported the theory that perceived competence is related to increased PA in adolescence [20,21]. However, some studies have shown the contradictory finding that actual FMS competence is a more important factor than perceived competence for promoting PA. For instance, Morgan et al. [22] and Slykerman et al. [23] found a positive link between actual FMS competence and MVPA in 5- to 9-year-old children, but no significant correlation between perceived competence and PA. Considering the mixed findings to date, further investigation is necessary in order to understand these relationships.

Stodden et al. [4] suggests the role of perceived competence as a mediator between actual FMS competence and PA, with children engaging in PA and developing higher perceptions of competence leading to greater FMS competence and more PA. This indicates that children who form a positive perception of competence based on their actual skill competence would be likely to engage consistently in a variety of physical activities [4,24]. A longitudinal study supports this notion, as perceived competence was reported to act as a mediator between children’s ball skills and later adolescent PA [21]. It is also noteworthy that the mechanisms between actual FMS competence, perceived competence, and PA could be different across child development: for instance, in early childhood, actual FMS competence and PA might not be strongly influenced by perceived competence due to children’s limited cognitive skills to distinguish their actual competency [4]. Theoretically, children’s actual competence and perceptions strengthen across their age and development, especially starting from middle childhood. This is because as children age, their cognitive ability to discern their own actual competence increases [25,26]. Interestingly, a recent meta-analysis did not find age was a moderator between actual and perceived motor competence [27], but the authors thought this might be because there were fewer studies with older samples. In addition, the mean ages of the study samples were used in the analysis and therefore age effects were already potentially leveled out within study sub-samples [27]. Nevertheless, middle childhood is hypothesized as a critical time to influence the trajectories between children’s FMS competence, PA behaviors, health-related fitness, and weight status across time [4], but there is only limited evidence about the role of perceived competence available in this age group. Although some previous studies examined the mechanisms of the mediational role of perceived competence on relationships between actual motor competence and PA, the age of the samples were younger children (age ranged from 4 to 6 years old; [28,29,30]) than our samples (>mean age 10 years old).

PA enjoyment, known as a significant factor in motivating children’s PA behaviors, has been explored in PA promotion-based intervention studies [2,31,32,33,34]. It is acknowledged that enjoyment plays a major role in increasing adherence to regular PA [35,36]. The relationship between perceived competence and enjoyment has been examined in previous studies. For instance, Cairney et al. [37] demonstrated that high perceived competence was significantly associated with high enjoyment in PE among school-aged children in a two-year longitudinal perspective. However, to our knowledge, no other studies have investigated perceived competence as a mediator between school-aged children’s ball skill competence and their PA and PA enjoyment.

Given that ball skill competence may be more critical to focus on than locomotor skill competence—as it has been related to determinants of subsequent PA and PA enjoyment [7]—the major purpose of this study was to investigate a mediating role of perceived competence on associations between actual ball skill competence and PA and PA enjoyment among school-aged children.

## 2. Materials and Methods

### 2.1. Participants and Procedures

Two hundred and ninety-four school-aged children (*M*_age_ = 10.96 ± 0.76; 51.7% boys) from a convenient sample participated in this study. They were recruited from three public elementary schools in the same school district in the U.S. southwestern region in 2013. The schools provided daily 30-min physical education (PE) classes taught by experienced PE teachers. The University Institutional Review Board (IRB), school administrators, and PE teachers permitted to conduct the study. Before collecting data, informed parental consent and child assent were collected.

Participants completed the self-reported questionnaires regarding perceived competence, PA, and PA enjoyment during their regular PE classes. Researchers assured students that their responses would be anonymous and remain confidential. Students’ actual ball skills were examined during three different PE classes based on the time limits. PE teachers assisted in coordinating the schedules and assessments. Before the data collection, research assistants engaged in multiple training sessions to understand the protocol of the study and to be masters of PE Metrics^TM^ assessment.

### 2.2. Measures

#### 2.2.1. Actual Ball Skill Competence

Recommended by PE teachers, three PE Metrics^TM^ items [38] were used to assess school-aged children’s actual ball skill competence in basketball (e.g., dribbling, passing, receiving), overhand throwing, and striking. PE Metrics^TM^ has been validated and used to examine school-aged students’ motor skills in PE [17,39]. Each skill has specific assessment criteria for performance and is rated according to four levels of the Likert scale (1 = *seldom*, 2 = *sometimes,* 3 = *usually*, 4 = *consistently*).

Before the evaluation, the graduate research assistants demonstrated each ball skill one time. Students performed (a) dribbling a basketball with control while moving, (b) passing a ball to a partner three times, and (c) catching a ball from a partner three times (total score ranges 0–12). For the overhand throwing task (form and accuracy to target), students demonstrated the overhand throwing of a ball to a large wall target for three trials (total score ranges 0–24). In addition, students were asked to show striking (form and continuous strike) through one trial with a plastic bat (total score ranges 0–8). Each motor skill was measured by two trained research assistants, and the results showed reliable and satisfactory intra-rater reliability using the two-way mixed effects model: intra-class correlation coefficient [ICC] for basketball = 0.85 (95% CI [0.81, 0.88]), overhand throwing = 0.93 (95% CI [0.92, 0.95]), and striking = 0.97 (95% CI [0.96, 0.97]).

#### 2.2.2. Perceived Competence

A twelve-item Perceived Competence Scale (PCS; [40]) was employed to examine children’s perceived competence. Children’s beliefs and predictions about three ball skills: basketball (dribbling, passing, and receiving), overhand throwing, and striking were measured, and the scale has been validated in a previous study involving similarly aged children to the current sample [17]. For instance, the sample question regarding striking asked, “*I feel confident in my ability to do striking*.” Each scale is composed of a 7-point Likert scale (7 = *very true* and 1 = *not true*). The four items in each skill were summed and produced a mean score, which indicates the perceived competence of each ball skill. An internal consistency test indicated acceptable reliability in basketball, overhand throwing, and striking (Cronbach’s alpha ranged from 0.83 to 0.90). In our sample, we examined the convergent validity of the PCS through confirmatory factor analysis (CFA) and found acceptable reliability and validity: average variance extracted (AVE) values (ranged from 0.51 to 0.68) and composite reliability (CR) values (ranged from 0.67 to 0.86 [41]).

#### 2.2.3. PA

The PA Questionnaire for Older Children (PAQ-C) was used to measure the level of PA during PE, recess, and lunchtime [42]. It is a 7-day recall questionnaire to assess MVPA. Participants responded to a 5-point Likert scale ranging from 5 (*High)* to 1 (*Low*). The PA scores were the mean of the sum scores from the nine items: 3 items of in-school PA in PE, recess, and lunch; 3 items of outside school PA right after school, and during evenings and weekends; 3 items of spare time PA/play (level and frequency of engagement). The PAQ-C shows adequate reliability and validity for measuring school-aged children PA [42,43], and also showed sufficient internal reliability among the participants in this study (α = 0.73).

#### 2.2.4. PA Enjoyment

Enjoyment of PA [44] was used to assess children’s PA enjoyment. Children responded to a 16-item PA enjoyment scale on a 5-point Likert scale ranging from 5 (*Agree a lot*) to 1 (*Disagree a lot*), with the stem “When I am active…” Two sample answers were “I enjoy it” and “I feel bored” (reverse item). The mean of a total score was used to indicate the participants’ enjoyment of PA. This measurement has been found to be a reliable and valid measure for elementary children [44], and Cronbach’s alpha coefficient was 0.85 in the present study.

### 2.3. Data Analysis

Statistical analyses were performed using SPSS 27.0 (IBM Corp., Armonk, NY, USA). No missing data and outliers were found, and all variables were normally distributed (see Table 1; skewness and kurtosis between −2 and +2; [45]). Descriptive statistics of all study variables, including the mean and standard deviation, were reported in Table 1. Pearson product-moment correlations were used to assess the associations among all variables. Lastly, structural equation modeling (SEM; AMOS 27.0) was conducted using maximum likelihood (MI) estimation to test the mediating role of perceived competence (a latent variable with three observed variables: perceived competences in basketball, overhand throwing, and striking) on the relationship between actual ball skill competence (a latent variable with three observed variables: basketball, overhand throwing, and striking) and PA and PA enjoyment (dependent variables), by controlling for sex and age, respectively.

The model fit was determined using multiple goodness-of-fit-indices [46], involving the chi-square (*χ*^2^), degree of freedom (df), comparative fit index (CFI), non-normed fit index (NFI), incremental fit index (IFI), root mean square error of approximation (RMSEA), and standardized root mean square residual (SRMR). The value of cut-offs of a good fit for CFI, NFI, and IFI is a score over 0.90, and exceeding 0.95 among the values indicates an excellent fit. A score of less than 0.06 in RMSEA and 0.08 in SRMR represents a well-fitting model [45]. In addition, the bootstrapping technique was applied to examine the statistical significance of the indirect effect (95% confidence intervals [CI]) of the SEM model [47].

## 3. Results

Table 1 indicates the descriptive statistics of the study variables. On average, the participants in this study received a 9.65 score (*SD* = 1.92) in basketball, a 15.99 score (*SD* = 3.74) in overhand throwing, and a 6.46 score (*SD* = 1.39) in striking among assessments of their actual ball skill competence. On average, the children acquired a 5.99 score (*SD* = 1.35) in basketball, a 5.91 score (*SD* = 1.30) in overhand throwing, and a 5.18 score (*SD* = 1.64) in striking among the measures for perceived ball skill competence. They received an average 3.24 score (*SD* = 0.66) in PA and 4.33 (*SD* = 0.65) in PA enjoyment.

The results of Pearson product-moment correlations (see Table 2) showed that actual ball skill competence indices were correlated (*rs* ranged from 0.36 to 0.41, *p* < 0.01). Actual ball skill competence indices were associated with most perceived ball skill competence indices (*rs* ranged from 0.16 to 0.27, *p* < 0.01), except between actual basketball skill competence and perceived striking skill competence, and between actual striking skill competence and perceived overhand throwing skill competence (*p* > 0.05). Only actual basketball skill competence was related to PA (*r* = 0.12, *p* < 0.05); no other correlations were found between actual ball skill competence indices and PA and PA enjoyment (*p* > 0.05). Perceived ball skill competences were correlated to one another (*rs* ranged from 0.36 to 0.56, *p* < 0.01). All perceived ball skill competence indices were associated with PA and PA enjoyment (*rs* ranged from 0.19 to 0.32, *p* < 0.01). A significant correlation between PA and PA enjoyment was also revealed (*r* = 0.34, *p* < 0.01).

Before structuring the mediation model, the direct paths from the actual ball skill competences and perceived ball skill competences with three latent variables (i.e., basketball, overhand throwing, and striking) to PA and PA enjoyment (dependent variables) were structured by controlling for sex and age, respectively (model not included). The goodness-of-fit indices supported a marginal fitting model (*χ*^2^/df = 27.04/16; CFI = 0.92; NFI = 0.88; IFI = 0.92; RMSEA = 0.08, SRMR = 0.04). Specifically, direct paths from actual ball skill competence to PA (*β* = 0.04; *p* = 0.57) and PA enjoyment (*β* = −0.05, *p* = 0.44) were nonsignificant. Further, there were positive path coefficients from perceived ball skill competence to PA (*β* = 0.29, *p* < 0.01) and PA enjoyment (*β* = 0.37, *p* < 0.01), and from PA enjoyment to PA (*β* = 0.24, *p* < 0.01). Thus, the relationships between actual ball skill competence and PA and PA enjoyment could be fully mediated by perceived ball skill competence [48].

**Figure 1:**
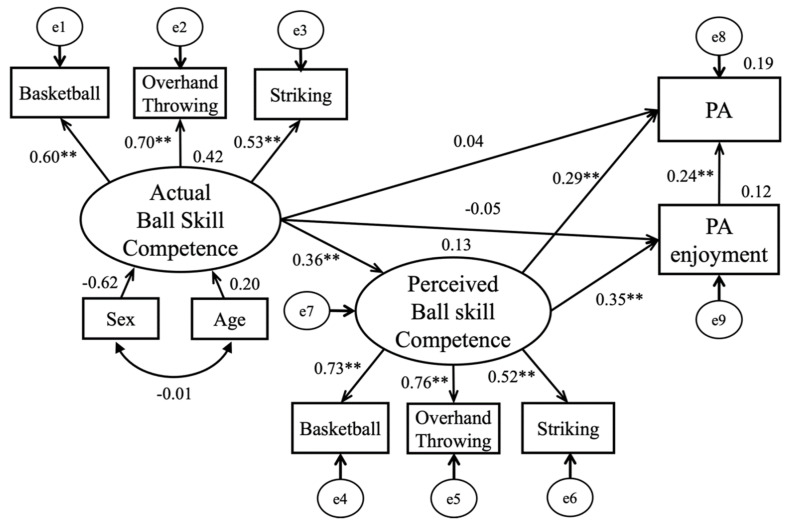
The Mediation Model with Standardized Path Coefficients. Note. ** *p* < 0.01.

Finally, the mediational model was structured by adding the direct path from actual ball skill competence to perceived ball skill competence and by controlling for sex and age. As shown in Figure 1, the goodness-of-fit indices suggested a well-fitting model (*χ*^2^/df = 52.03/32; CFI = 0.96; NFI = 0.90; IFI = 0.96; RMSEA = 0.05, SRMR = 0.04). Specifically, actual ball skill competence was directly and positively associated with perceived ball skill competence (*β* = 0.36, *p* < 0.01), explaining 13% of the variance, which in turn significantly predicted PA (*β* = 0.29, *p* < 0.01) and PA enjoyment (*β* = 0.35, *p* < 0.01). It was also found that PA enjoyment partially mediated the relationship between perceived competence and PA. The statistically significant indirect effect of actual ball skill competence on PA (*β* = 0.14; 95% CI [0.052, 0.153], *p* < 0.01) and PA enjoyment (*β* = 0.15; 95% CI [0.042, 0.128], *p* < 0.01) was supported based on the bootstrapping tests, respectively. The model accounted for 19% and 12% of the variances in PA and PA enjoyment, respectively.

## 4. Discussion

Given that school-aged children do not achieve daily PA suggestions and are not physically active on a regular basis [3], the main purpose of this study was to investigate the relationships among elementary school students’ actual ball skill competence, perceived competence, PA, and PA enjoyment. Using the SEM technique, the importance of a mediating role of perceived competence on relationships between actual ball skill competence and children’s PA and PA enjoyment was tested and confirmed. As suggested by the hypothesized model, the results did not indicate a direct effect of actual ball skill competence on PA and PA enjoyment in our sample. Based on the final mediation model, actual ball skills significantly predicted school-aged children’s perceived competence, which positively affected their PA and PA enjoyment. This study provided meaningful insights about the importance of promoting perceived competence to increase PA and PA enjoyment among school-aged children.

The findings suggested that perceived ball skill competence via actual ball skill competence promoted PA and PA enjoyment. For example, school-aged children with higher perceived competence, affected by actual motor skill competence, were more likely to be physically active and had greater enjoyment of PA than those with lower perceived competence. Previous studies [22,23,28,49], however, have found a significant role and direct effect of actual ball skill competence, rather than perceived competence, on children’s PA (age range 3–9 years old). This inconsistent finding might be due to the older age group in our sample (>mean age = 10 years old), indicating that PA participation may be mainly driven by perceived competence as school-aged children approach adolescence [20,21]. This supports Stodden et al.’s conceptual model [4], which hypothesized that a mediating role of percieved motor competence on pathways between actual motor skills and PA could differ by age changes.

Consistent with the findings regarding a similar age group examining underserved minority Hispanic children [17], our findings supported the essential role of perceived competence as a mediating factor to benefit students’ PA. In addition, similar to previous research findings, school-aged children with higher perceived competence have a greater enjoyment of PA [37]. The findings of the present study support Stodden et al.’s [4] conceptual model, which showed a mediating role of perceived competence between actual motor skill competence and PA engagement in school-aged children. To enhance perceived ball skill competence for school-aged children, it is suggested that supportive skill-related environments in school settings should be provided, such as offering sufficient equipment for students to learn and play [17,32]. The present study also found a second indirect effect of PA enjoyment on the path between perceived ball skill competence and PA. Both perceived competence and PA enjoyment were important correlates of PA in a previous study [36]. Children were more likely to experience enjoyment once they were competent in different ball skills, which in turn promoted their PA participation. Echoing the suggestions of previous studies [5,7,50], PE teachers should aim at improving students’ ball skills with a need-supportive environment that encourages them to feel competent to engage in various types of games and that promotes PA enjoyment.

Although the present study represents an initial research effort to utilize the SEM to test the mediating role of perceived competence on the relations between actual motor skill competence and PA and PA enjoyment, this cross-sectional study is limited in being able to determine the causal relationships. Future studies need to examine multiple time points to provide evidence of the important role of perceived competence that could contribute to building practical applications. In addition, the inability to explain known contributors to children’s actual ball skill competence, PA, and PA enjoyment limits our findings because there may have been other factors that impacted those variables. Our study limitations also include not measuring locomotor skills and other ball skills, including kicking and catching; therefore, future research needs to comprehensively examine actual locomotor and ball skill competences. We also acknowledge the limitation that there are other factors beyond perceived competence that can contribute to children’s PA and PA enjoyment, such as motivation [16], weight status, and fitness [51]. Measuring PA with a self-reported assessment is another limitation of this study. The PAQ-C has shown reliability and validity in measuring self-reported PA, and so a questionnaire was chosen in this study. However, an objective PA tool (e.g., pedometers and accelerometers) may be necessary to explore the relationship to objective PA in future studies. The use of a perception tool in which the skill items were completely aligned to the tool used to measure actual skill is a strength of the study [52], even though this tool was not widely validated. Even so, correlations between actual and perceived skill were not high—suggesting other influencing factors may have been involved [27].

In conclusion, the present study provides empirical evidence that perceived competence may play a role as a mediator in the relationships between actual ball skill competence and PA and PA enjoyment among school-aged children. It is noted that PE teachers may need to focus on enhancing students’ confidence in ball skills to encourage them to engage in regular PA and to improve their enjoyment of PA. Providing a supportive PA environment and sufficient equipment is recommended to encourage children to develop ball skill competence, which leads to increased perceived competence.

## Figures and Tables

**Table 1:** Descriptive statistics of study variables (N = 294).

Variables	Range	*M*	*SD*	Skewness	Kurtosis
**Actual ball skill competence**					
Basketball	5.50–12	9.65	1.92	−0.44	−0.96
Overhand Throwing	6.50–24	15.99	3.74	0.26	−0.65
Striking	2.50–8	6.46	1.39	−1.01	0.02
**Perceived ball skill competence**					
Basketball	1–7	5.99	1.35	−1.45	1.41
Overhand Throwing	1–7	5.91	1.30	−1.45	1.68
Striking	1–7	5.18	1.64	−0.81	−0.15
**PA**	1.67–4.81	3.24	0.66	−0.07	−0.59
**PA enjoyment**	1.75–5	4.33	0.65	−1.31	1.62

Note. *M* = Mean, *SD* = Standard deviation.

**Table 2:** Correlations among the study variables (N = 294).

Variables	1	2	3	4	5	6	7	8
1. Basketball	-							
2. Overhand Throwing	0.41 **	-						
3. Striking	0.36 **	0.36 **	-					
4. PC-Basketball	0.16 **	0.27 **	0.16 **	-				
5. PC-Overhand Throwing	0.16 **	0.21 **	0.08	0.56 **	-			
6. PC-Striking	0.11	0.17 **	0.17 **	0.36 **	0.39 **	-		
7. PA	0.12 *	0.02	0.10	0.19 **	0.32 **	0.27 **	-	
8. PA enjoyment	0.05	0.02	0.03	0.27 **	0.24 **	0.25 **	0.34 **	-

Note. PC = perceived competence; ** *p* < 0.01, * *p* < 0.05.

## Data Availability

The data presented in this study are available on request from the corresponding author. The data are not publicly available due to consent provided by participants.
